# Sarcopenic Obesity Phenotype Index (SOP*i*): A Population‐Based Study

**DOI:** 10.1002/jcsm.70099

**Published:** 2025-10-15

**Authors:** Elizabeth Benz, Alexandre Pinel, Christelle Guillet, Frederic Capel, Bruno Pereira, Dimitris Rizopoulos, Alfonso J. Cruz‐Jentoft, Doris Eglseer, Eva Topinkova, Rocco Barazzoni, Lorenzo M. Donini, Fernando Rivadeneira, Marinka Steur, Trudy Voortman, Peter J. M. Weijs, Josje D. Schoufour, Yves Boirie

**Affiliations:** ^1^ Human Nutrition Unit Clermont Auvergne University, Institut National de Recherche Pour l'Agriculture, l'Alimentation et l'Environnement, Centre de Recherche en Nutrition Humaine Clermont‐Ferrand France; ^2^ Department of Epidemiology Erasmus MC, University Medical Center Rotterdam Rotterdam the Netherlands; ^3^ Unit of Biostatistics Clermont‐Ferrand University Hospital Clermont‐Ferrand France; ^4^ Department of Biostatistics Erasmus MC, University Medical Center Rotterdam Rotterdam the Netherlands; ^5^ Servicio de Geriatria Hospital Universitario Ramon y Cajal (IRYCIS) Madrid Spain; ^6^ Institute of Nursing Science Medical University of Graz Graz Austria; ^7^ Department of Geriatrics, First Faculty of Medicine Charles University and General University Hospital, Prague, Czech Republic, and University of South Bohemia, Faculty of Health and Social Sciences Ceske Budejovice Czech Republic; ^8^ Department of Medical, Surgical and Health Sciences University of Trieste Trieste Italy; ^9^ Department of Experimental Medicine, Section of Medical Pathology, Endocrinology and Human Nutrition Sapienza University of Rome Rome Italy; ^10^ Department of Internal Medicine Erasmus University MC Rotterdam the Netherlands; ^11^ Division of Human Nutrition and Health Wageningen University Wageningen the Netherlands; ^12^ Department of Nutrition and Dietetics, Faculty of Health, Sport and Physical Activity Amsterdam University of Applied Sciences Amsterdam the Netherlands; ^13^ Department of Nutrition and Dietetics, Amsterdam University Medical Centers, Amsterdam Public Health Institute VU University Amsterdam the Netherlands; ^14^ Clinical Nutrition Department Clermont‐Ferrand University Hospital Clermont‐Ferrand France

**Keywords:** phenotype, population‐based study, sarcopenia, sarcopenic obesity, survival

## Abstract

**Background:**

Sarcopenic obesity (SO) is a clinical condition defined by the coexistence of high body fat mass and low muscle function and mass, which increases the risk of adverse health outcomes, including disability and mortality. Early detection and frequent monitoring of SO are essential for preventive interventions and management strategies. The current binary approach for SO diagnosis is limited in capturing the spectrum of SO or its progression over time. The main objective of this study was to develop a continuous SOP*i* that integrates diagnostic criteria such as muscle function and body composition. We aimed to evaluate the association between SOP*i* and all‐cause mortality, to identify baseline‐related factors with SOP*i* and to assess changes in the SOP*i* over time.

**Methods:**

Participants from the Rotterdam Study with baseline and follow‐up measures of handgrip strength (HGS), dual‐energy X‐ray absorptiometry‐measured appendicular lean mass index (ALM/kg) and body fat percentage (BF%) were included. SOP*i* was calculated as a sex‐specific equation integrating z‐scores (Z) of (BF%)—(HGS)—(ALM/kg). Cox regression and multivariable linear regression models were fitted to evaluate mortality risk and associated factors with SOP*i*, respectively. Subgroup analysis of SOP*i* changes was performed by linear mixed‐effects models.

**Results:**

In the total population (*n* = 5888, age 69.5 ± 9.1 years, BMI 27.5 ± 4.3 kg/m^2^, 56.8% females) and over the 9.9‐year median follow‐up period, 1538 (26.1%) participants died. Each standard deviation (SD) increase in sex‐specific SOP*i* was associated with a 10% higher risk of premature death (HR = 1.10 [95%CI: 1.07; 1.13]). Thirteen factors were associated with high SOP*i*, such as reduced physical activity, higher triglyceride‐glucose index, HOMA‐IR, systemic inflammation, osteopenia, hypertension, liver steatosis, asthma, coronary heart disease, oral corticosteroid use, lower protein intake, lower quality of life and lower educational status.

In participants with obesity, lower physical activity and/or insulin resistance (*n* = 1682), a significantly higher and faster increase in SOP*i* was observed compared to participants without these factors (males: β = 2.63 [95%CI: 2.22; 3.03]; females: β = 2.90 [95%CI: 2.58; 3.23]).

**Conclusion:**

SOP*i* is a significant predictor of premature death and can identify associated factors, particularly useful among persons at risk of SO. SOP*i* is higher and increases faster in individuals with specific phenotypes. SOP*i* integrates prognosis information, which could be used as a risk indicator and for prevention of SO.

## 1 Introduction

Sarcopenic obesity (SO) is clinically diagnosed as an alteration in body composition (low muscle mass and high body fat percentage) and muscle function and is associated with an increased risk of morbidity, disability and premature mortality [1, 2, 3, 4, 5, 6, 7]. Differential changes in the phenotypic criteria of SO have been generally gone undetected in terms of their impact on adverse health outcomes [1, 2, 3]. Based on recent definitions, SO prevalence ranges from 0.8% to 23%, depending on the cut‐offs of diagnostic criteria, muscle mass assessment techniques and population settings [3, 8, 9, 10, 11, 12, 13, 14]. While SO is predominantly prevalent among older people, it can also manifest at a younger age [15]. The ESPEN/EASO consensus has made great progress by proposing cut‐off values to identify low muscle mass and function to define people with obesity with apparent SO [1]. Similarly, the Lancet Diabetes & Endocrinology Commission has recently published a new definition and diagnostic framework for obesity, encompassing both preclinical and clinical aspects [16]. Nevertheless, it seems that there is a group of persons who are at risk of adverse health outcomes without clinically present SO [3]. As a result, the limitations of these cut‐off values in detecting persons with both low muscle function, low muscle mass and high fat mass might overlook a risk of SO [17]. Furthermore, dichotomous outcomes are restricted to examining transitions or progression over time [18]. Therefore, utilizing a continuous scale for SO might contribute to clarifying the differences in the contemporaneous categories among various populations, as well as identifying risk groups.

Specifically, the current definition of SO may not include adults exhibiting early signs of risk factors for SO, potentially delaying therapeutic or preventive interventions [19, 20, 21, 22]. SO might be influenced by multiple shared factors and pathophysiological pathways that affect both sarcopenia and obesity, including age, sedentary lifestyle, lack of muscle exercise, hormone levels, nutritional status, insulin resistance, systemic inflammation and others [14, 23, 24, 25, 26, 27]. A recent literature review described five main factors associated with SO (i.e., insulin resistance, dyslipidaemia, exercise training, inflammation and hypertension) from a total of 39 [28]. Identifying key factors associated not only with SO as dichotomous outcomes but also with earlier and/or continuous measures of SO risk could contribute to creating targeted interventions to prevent SO and enhance our understanding of the underlying biological pathways involved.

This study aimed (1) to develop a continuous sex‐specific sarcopenic obesity phenotype index (SOP*i*), (2) to analyse the association of SOP*i* with the risk of premature all‐cause mortality in persons with and without current diagnostic criteria of SO, (3) to assess a comprehensive set of clinical and lifestyle‐related factors associated with SOP*i* and (4) to examine how SOP*i* changes over time.

## 2 Methods

### 2.1 Study Design and Participants

This cross‐sectional and longitudinal study was embedded within the Rotterdam Study (RS), a prospective cohort study from the Netherlands ongoing since 1990 [29]. The RS has been designed to assess the occurrence and risk factors of chronic diseases in middle‐aged and older people. Currently, it includes almost 18 000 participants aged ≥ 40 years, residing in Ommoord, a district of Rotterdam [29]. All participants who visited the research centre from 1 March 2009 to 1 June 2014 were included in our study, while those who had no reliable or available measurements of handgrip strength (HGS) and dual‐energy X‐ray absorptiometry (DXA) scan were excluded. Subgroup analyses incorporated follow‐up data on repeated measurements of SO for persons participating between 1 May 2014 and 1 May 2016.

The RS has been approved by the Medical Ethics Committee of the Erasmus Medical Center, Rotterdam (registration number MEC 02.1015) and by the Dutch Ministry of Health, Welfare and Sport (Population Screening Act WBO, licence number 1071272‐159521‐PG). According to the Declaration of Helsinki [30], all included participants provided written informed consent to participate in the study and to obtain information from their treating physicians.

This study adheres to the Strengthening the Reporting of Observational Studies in Epidemiology (STROBE) reporting guideline.

### 2.2 Assessments

#### 2.2.1 Assessment of the Diagnostic Criteria of Sarcopenic Obesity

All participants underwent assessment of the components of SO during a visit to the research centre, following the ESPEN/EASO Consensus (Table S1) [1, 2]. The diagnosis process initiates with the assessment of muscle function (first criterion), measured as the maximum value of HGS obtained from three trials performed in the nondominant hand. A single trained examiner conducted the handgrip test using a hydraulic hand dynamometer (Fabrication Enterprises Inc., White Plains, NY, USA).

Dual X‐ray absorptiometry with a total body‐beam densitometer (iDXA, GE Lunar Corp, Madison, WI, USA) was employed to evaluate body composition, encompassing total mass, lean and fat mass. The scans were analysed using enCORE software V13.6, which provided measurements across predefined body regions of interest: the head, trunk, arms and legs. Appendicular lean mass (ALM), now referred to as appendicular lean soft tissue mass (ALST), denotes the sum of the lean tissue from the upper and lower limbs [31]. The ALM index (second criterion) was defined as ALM divided by body weight (kg). Body fat percentage (BF%) (third criterion) was calculated as total body fat mass divided by body weight multiplied by 100. Body mass index (BMI) was calculated as weight in kilograms divided by height in meters squared (kg/m^2^). Obesity was defined as a BMI ≥ 27 kg/m^2^, as this threshold can accurately predict BF% in older people [32].

#### 2.2.2 Assessment of Clinical and Lifestyle Factors

A total of 32 clinical and lifestyle factors were identified based on previous studies relating to their potential association with SO [28]. All clinical and lifestyle‐related factors were assessed at baseline through biological sample analyses, interviews, questionnaires, physical examinations and testing at the research centre, as well as through linkage with medical and pharmacy records.

We included 19 clinical factors such as homeostasis model assessment of insulin resistance score (HOMA‐IR) [fasting glucose (mmol/L) × fasting insulin (m UI/L)/22.5], triglycerides‐glucose index (TyG) [ln (fasting triglycerides) (mg/dL) × (fasting glucose) (mg/dL)/2]; high HOMA‐IR and TyG were defined as ≥ 2.0 and ≥ 4.68, respectively [33, 34]. Dyslipidaemia was defined as HDL < 1.0 mmol/L in males and < 1.3 mmol/L in females [35]. Systemic immune‐inflammation index (SII) was calculated from the platelets (P; ×10^9^/L), granulocytes, as a proxy for neutrophils (N; ×10^9^/L) and lymphocytes (L; ×10^9^/L), using the formula: SII = P× N/L [36]. High SII was defined as sex‐specific median. Full blood cell counts were performed on a COULTERS Act. T diff2 Hematology Analyzer (Beckman Coulter, San Diego, CA, USA).

Other clinical factors included were estimated glomerular filtration rate (eGFR) (calculated with calibrated creatinine values using the equation from the Chronic Kidney Disease Epidemiology Collaboration), predicted values of forced expiratory volume in 1 s, total bone mineral density (BMD) and the use of medications such as oral corticosteroids (OCS) and antidepressive therapy (if one OCS/antidepressive prescription was delivered before the DXA scan date). Ten comorbidities were incorporated into the clinical factors: type 2 diabetes (T2D) (fasting plasma glucose level ≥ 7 mmol/L, or a nonfasting plasma glucose level ≥ 11.1 mmol/L or the use of blood glucose‐lowering medication), hypertension (HT) (systolic blood pressure ≥ 140 mmHG or diastolic blood pressure ≥ 90 mmHg or use of antihypertensive medication), osteopenia or osteoporosis (bone mineral density *t*‐score between −1.0 to −2.5 or equal to −2.5, respectively), cancer (clinical‐based), coronary heart diseases (CHD) (myocardial infarction, coronary bypass grafting or percutaneous coronary intervention), liver steatosis, non‐alcoholic fatty liver disease (NAFLD), metabolic associated liver disease (MAFLD) (clinical‐based), chronic respiratory diseases such as chronic obstructive pulmonary disease (COPD) and asthma (clinical‐ and spirometry‐based), and depressive symptoms (Center for Epidemiologic Studies Depression scale).

Moreover, 13 lifestyle factors were included: physical activity (PA) (assessed using a validated adapted version of the Longitudinal Aging Study Amsterdam Physical Activity Questionnaire and expressed in metabolic equivalent of task (MET)‐hours per week) [37], total calorie intake, fibre intake, fat intake, carbohydrate intake, daily protein intake (dichotomized at 1.0 g/kg) (calculated from Food‐frequency questionnaires and standardized for energy intake [g/kcal] and body weight) [38], health‐related quality of life (HRQL) was measured using the Dutch version of the EuroQol‐5 dimensions, educational status (primary, lower, intermediate or higher education according to the UNESCO classification), sleep quality (Pittsburgh Sleep Quality index), alcohol intake (g/d), smoking status (current, past or never), smoking pack‐years (dichotomized at 20 pack‐years), retirement status (if receipt of an official retirement pension [statutory retirement due to age ≥ 65 years], early retirement and renter or who is not retired but he/she is not working and living off the interest of their real assets [i.e., properties]).

#### 2.2.3 Assessment of All‐Cause Mortality

General practitioners and the central registry of the Municipality of Rotterdam provided mortality data information. Follow‐up time started at the date of the DXA scan, assessed between 1 March 2009 and 1 June 2014 and ended at the date of death or the end of the study (20/10/2022), whichever came first.

### 2.3 Statistical Analyses

First, to formulate a continuous and standardized index reflecting SO level, we calculated z‐scores for each of the three phenotypic components of SO: (1) body fat percentage (BF%), (2) HGS and (3) ALM adjusted by body weight (ALM/kg). Z‐scores were calculated using sex‐specific means (μ), and standard deviations (*σ*) derived from the total population study of the RS. A z‐score is defined as Z = (χ‐μ)/σ, where Z = z‐score, χ is the observed measurement, μ is the mean and σ is the standard deviation of HGS, ALM/kg or BF% of the RS.

These z‐scores were combined into a SOP*i* as follows:

$$\begin{array}{c} \text{SOPi}=\left(\frac{\mathrm{BF}\%-\mathrm{\mu BF}\%\mathrm{RS}}{\mathrm{\sigma BF}\%\mathrm{RS}}\right)-\left(\frac{\mathrm{HGS}-\text{μHGSRS}}{\text{σHGSRS}}\right)-\left(\frac{\frac{\mathrm{ALM}}{\mathrm{kg}}-\frac{\text{μALMRS}}{\mathrm{kg}}}{\frac{\text{σALMRS}}{\mathrm{kg}}}\right) \end{array}$$

Higher SOP*i* values correspond to an elevated level of SO. Baseline *μ* and *σ* values were used to calculate SOP*i* at follow‐up, ensuring consistency and minimizing variability from shifting reference values over time. An increase in SOP*i* over time indicates a deterioration of the SO parameters. Additionally, quartiles of SOP*i* were calculated to enhance the interpretability of the results.

Second, probabilities for any cause of death were plotted using Kaplan–Meier curves in all populations, as well as in participants without any clinical diagnostic criteria of SO and in participants with at least one clinical criterion for SO. Multivariable Cox regression models were fitted to estimate the hazard ratios (HRs) and 95% confidence intervals (CIs) for all populations, and for participants without any diagnostic criteria for SO. Model 1 was unadjusted, Model 2 was adjusted for sex and age and Model 3 was further adjusted for BMI. The proportional hazard assumption was studied through Schoenfeld's test and plotting residuals.

Third, a manual combined forward‐afterward strategy was followed in linear regression models to identify the factors associated with SOP*i*. In a first step, the association of 32 preselected factors with the SOP*i* was analysed individually, adjusted for age and sex (Model 1). Statistically significant factors associated with SOP*i* in model 1 (*p* < 0.05) were examined in two multivariable models: one combining the significant clinical factors (Model 2) and another combining the lifestyle factors (Model 3). In a second step, factors that retained significance within each multivariable model were analysed together in a single multivariable model (Model 4). Last, all significant factors from Model 4 with a variance inflation factor (VIF) < 2.0 were incorporated into the final multivariable model (Model 5) and displayed in a heat map.

Fourth, to examine the SOP*i* changes over time, repeated measurements of sex‐specific SOP*i* were analysed by linear mixed models. A subgroup of participants with complete baseline and follow‐up measurements of SOP*i* was categorized based on the presence/absence of obesity, as well as clinical/lifestyle factors associated with SOP*i* and its three components. The study groups were participants (1) without obesity and 0 factors (reference); (2) without obesity and 1 or more factors; (3) with obesity and 0 factors; and (4) with obesity and 1 or more factors. Age served as the time scale, incorporating random intercepts and slopes, alongside an unstructured variance–covariance matrix and adjusted for age at baseline. In addition, we examined whether the assumptions for residual terms were met by utilizing scatter and Q‐Q plots of the marginal and conditional residuals.

Statistical analyses were carried out using RStudio version 4.3.3 (Foundation for Statistical Computing, Austria), using the packages *survival*, *ROCR* and *nlme*. A *p*‐value of < 0.05 was considered to indicate statistical significance in all analyses.

#### 2.3.1 Sensitivity Analyses

We conducted different sensitivity analyses. Firstly, we tested the potential nonlinear effect of SOP*i* on mortality by employing restricted splines with boundary knots at the 5th and 95th percentiles within the Cox models. Secondly, we tested the diagnostic performance of SOP*i* for all‐cause mortality through receiver‐operating characteristic curve analysis (ROC). Thirdly, percentiles of SOP*i* and its components within the total population were described for age and sex. Fourthly, to enhance the accuracy of DXA measurements, participants with severe obesity (BMI ≥ 35 kg/m^2^, weight > 136 kg or height > 195.6 cm) were excluded.

## 3 Results

**FIGURE 1:**
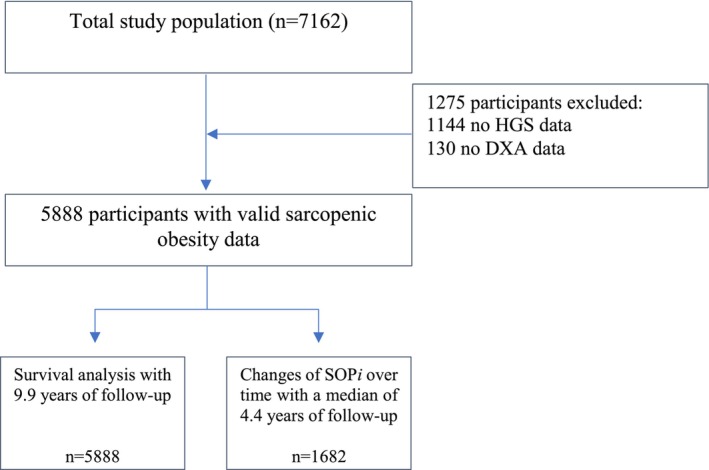
Flow chart and analysis process of the study population.

A total of 7162 participants were assessed at baseline, with 5888 providing complete data on body composition and HGS (Figure 1). The total population had a mean age of 69.5 years (SD = 9.1) and a mean BMI of 27.5 kg/m^2^ (SD = 4.3); 3343 (56.8%) were female, and 2545 (43.2%) were male. SOP*i* was calculated based on the following formula:

$$\begin{array}{c} \begin{array}{c} \mathrm{SOP}i\;\text{Male}=\left(\frac{\mathrm{BF}\%-31.06\%}{5.53}\right)-\left(\frac{\mathrm{HGS}-36.47\mathrm{kg}}{8.93}\right)-\left(\frac{\frac{\mathrm{ALM}}{\mathrm{kg}}-30.40\mathrm{kg}}{3.00}\right) \end{array} \end{array}$$

$$\begin{array}{c} \mathrm{SOP}i\;\text{Female}=\left(\frac{\mathrm{BF}\%-39.32\%}{6.08}\right)-\left(\frac{\mathrm{HGS}-21.82\mathrm{kg}}{5.80}\right)-\left(\frac{\frac{\mathrm{ALM}}{\mathrm{kg}}-25.14\mathrm{kg}}{2.65}\right) \end{array}$$

SOP*i* ranged between −10.1 (less SO) and 8.29 (more SO). All participants were categorized into SOP*i* quartiles. Table 1 presents demographic, anthropometric, clinical and lifestyle factors.

**TABLE 1:** Characteristics of the study population^a^.

Factors	All population	SOP*i* Quartile 1	SOP*i* Quartile 2	SOP*i* Quartile 3	SOP*i* Quartile 4
	*n* = 5888	*n* = 1472	*n* = 1472	*n* = 1472	*n* = 1472
	z = [−10.10; 8.29]	z = [−10.10; −1.48]	z = [> −1.48; < 0.09]	z = [> 0.09; < 1.58]	z = [> 1.58; 8.29]
Age (years)	69.5 ± 9.06	64.9 ± 7.80	68.2 ± 8.46	70.9 ± 8.63	73.9 ± 8.75
BMI, kg/m^2^	27.5 ± 4.26	24.6 ± 3.02	26.6 ± 3.12	28.2 ± 3.54	30.6 ± 4.66
Sex, *n* (%)					
*Male*	2545 (43.2)	656 (44.6)	630 (42.8)	627 (42.6)	632 (42.9)
*Female*	3343 (56.8)	816 (55.4)	842 (57.2)	845 (57.4)	840 (57.1)
(a) Clinical factors					
1. TyG index	4.69 ± 0.25	4.60 ± 0.24	4.67 ± 0.25	4.73 ± 0.25	4.77 ± 0.25
High TyG (≥ 4.68), *n* (%)	2806 (47.7)	476 (32.3)	666 (45.2)	788 (53.5)	876 (59.5)
2. HOMA‐IR	2.59 [1.73; 4.03]	1.92 [1.35; 2.78]	2.44 [1.64; 3.68]	2.89 [1.98; 4.43]	3.42 [2.30; 5.33]
High HOMA‐IR (≥ 2.0)	3863 (65.6)	690 (46.9)	916 (62.2)	1069 (72.6)	1188 (80.7)
3. SII index	461 [342; 629]	438 [329; 591]	445 [333; 596]	461 [350; 636]	498 [371; 682]
High SII (≥ 460)	2887 (49.0)	665 (45.2)	680 (46.2)	720 (48.9)	822 (55.8)
4. eGFR (< 60 mL/min/1.73m^2^), *n* (%)	857 (14.6)	119 (8.1)	171 (11.6)	242 (16.4)	325 (22.1)
5. Dyslipidaemia, *n* (%)	1221 (20.7)	208 (14.1)	275 (18.7)	353 (24.0)	385 (26.2)
6. FEV_1_% pred	97.3 ± 18.5	102.0 ± 17.4	99.1 ± 17.4	96.7 ± 18.4	91.3 ± 19.0
7. Total BMD, g/cm^2^	1.11 ± 0.14	1.12 ± 0.15	1.11 ± 0.14	1.11 ± 0.14	1.10 ± 0.14
*Medication use, n (%)*					
8. OCS	153 (2.6)	19 (1.3)	28 (1.9)	37 (2.6)	69 (4.7)
9. Antidepressive therapy	274 (4.7)	39 (2.6)	78 (5.3)	73 (5.0)	84 (5.7)
*Comorbidities, n (%)*					
10. T2D	873 (14.8)	103 (7.0)	192 (13.0)	250 (17.0)	328 (22.3)
11. Osteopenia	3190 (54.2)	809 (55.0)	800 (54.3)	780 (53.0)	801 (54.4)
12. Osteoporosis	386 (6.3)	88 (6.0)	75 (5.1)	92 (6.3)	113 (7.7)
13. HT	4304 (73.1)	793 (53.9)	1041 (70.7)	1175 (79.8)	1295 (88.0)
14. COPD	779 (13.2)	212 (14.4)	193 (13.1)	170 (11.5)	204 (13.9)
15. Asthma	439 (7.5)	84 (5.7)	90 (6.1)	116 (7.9)	149 (10.1)
16. Liver steatosis	2051 (34.8)	253 (17.2)	427 (29.0)	610 (41.4)	761 (51.7)
17. CHD	524 (8.9)	79 (5.4)	104 (7.1)	150 (10.2)	191 (13.0)
18. Depression symptoms	513 (8.7)	120 (8.2)	95 (6.5)	118 (8.0)	180 (12.2)
19. Cancer	554 (9.4)	105 (7.1)	127 (8.6)	145 (9.9)	177 (12.0)
20. NALFD	1543 (26.2)	175 (11.9)	308 (20.9)	465 (31.6)	595 (40.4)
21. MALFD	1974 (33.5)	223 (15.1)	410 (27.9)	590 (40.1)	751 (51.0)
(b) Lifestyle factors					
1. PA, METh/week	11.5 [5.0; 22.0]	15.0 [7.0; 25.0]	12.8 [5.6; 23.9]	10.4 [4.5; 20.6]	7.5 [3.5; 18.0]
*Low PA (< 11.5), n (%)*	2576 (43.8)	541 (36.8)	607 (41.2)	696 (47.3)	732 (49.7)
2. Total calorie intake, kg	2140 ± 698	2320 ± 690	2190 ± 683	2090 ± 699	1960 ± 668
3. Daily protein intake, g/kg	1.08 ± 0.38	1.24 ± 0.39	1.12 ± 0.36	1.03 ± 0.36	0.92 ± 0.31
*< 1.0 g/kg, n (%)*	1053 (17.9)	117 (7.9)	193 (13.1)	313 (21.3)	430 (29.2)
4. Fibre intake (g)	27.5 ± 11.2	30.5 ± 11.5	28.0 ± 10.8	26.6 ± 11.2	24.6 ± 10.5
5. Fat intake (g)	77.4 ± 35.6	82.8 ± 32.5	79.4 ± 36.2	75.6 ± 36.1	71.8 ± 36.5
6. Carbohydrate intake (g)	241 ± 86.9	265 ± 88.8	246 ± 83.8	236 ± 86.1	218 ± 82.1
7. *Low HRQL (< 0.8), n (%)*	1055 (17.9)	166 (11.3)	196 (13.3)	274 (18.6)	419 (28.5)
8. Educational status, *n* (%)					
*Primary education*	489 (8.3)	81 (5.5)	101 (6.9)	136 (9.2)	171 (11.6)
*Lower education*	2307 (39.2)	471 (32.0)	594 (40.4)	593 (40.3)	649 (44.1)
*Intermediate education*	1739 (29.5)	436 (29.6)	416 (28.3)	451 (30.6)	436 (29.6)
*Higher education*	1291 (21.9)	466 (31.7)	346 (23.5)	275 (18.7)	204 (13.9)
9. Low sleep quality (tertile 3), *n* (%)	1895 (32.2)	394 (26.8)	454 (30.8)	510 (34.6)	537 (36.5)
10. Alcohol intake, g	8.36 ± 8.50	8.52 ± 8.16	8.57 ± 8.42	8.11 ± 8.48	8.25 ± 8.96
11. Smoking status, *n* (%)					
*Never*	2021 (34.3)	537 (36.5)	489 (33.2)	512 (34.8)	483 (32.8)
*Past*	3154 (53.6)	689 (46.8)	793 (53.9)	799 (54.3)	873 (59.3)
*Current*	713 (12.1)	246 (16.7)	190 (12.9)	161 (10.9)	116 (7.9)
12. Smoking pack‐year	5.0 [0;10]	3.4 [0;19]	4.2 [0;21.8]	5.0 [0;25]	7.0 [0;27]
13. Retirement status, *n* (%)	3546 (60.2)	640 (43.5)	859 (58.4)	966 (65.6)	1081 (73.4)

*Note:* Number (percentage) of missing values per variable as follows: HOMA‐IR: 120 (2.0%), TyG: 112 (1.9%); SII: 99 (1.7%); eGFR: 113 (1.9%); dyslipidaemia: 112 (1.9%); FEV_1_: 582 (9.9%); T2D: 110 (1.9%); cancer: 582 (9.9%); MAFLD: 138 (2.3%); OCS: 2 (0.0%); osteopenia/osteoporosis: 132 (2.2%); HT: 1 (0.0%); COPD/asthma: 583 (9.9%); liver steatosis: 134 (2.3%); CHD: 65 (1.1%); depressive symptoms: 36 (0.6); PA: 747 (12.7%); daily energy/protein/fibre/fat/carbohydrate intake: 1175 (20%); HQOL: 11 (0.2%); education: 62 (1.1%); sleep quality: 251 (4.3%); alcohol intake 1175 (20%); smoking pack‐years: 14 (0.2%); retired status: 228 (3.9%).

Abbreviations: BMI, body mass index (calculated as weight in kilograms divided by height in meters squared); CHD, coronary heart diseases; COPD, chronic obstructive pulmonary disease; eGFR, estimated glomerular filtration rate; HOMA‐IR, Homeostatic Model Assessment for Insulin Resistance; HRQL, health‐related quality of life; HT, hypertension; MALFD, metabolic associated liver disease; MET, metabolic equivalent of task; NALFD, non‐alcoholic fatty liver disease; OCS, oral corticosteroid use; PA, physical activity; SII, systemic immune‐inflammation index; T2D, Type 2 diabetes; Total BMD, total bone mineral density; TyG, triglycerides‐glucose index.

^a^ Data are presented as number (percentage) of participants, mean ± SD or median (interquartile range).

### 3.1 SOP*i* and All‐Cause Mortality

**FIGURE 2:**
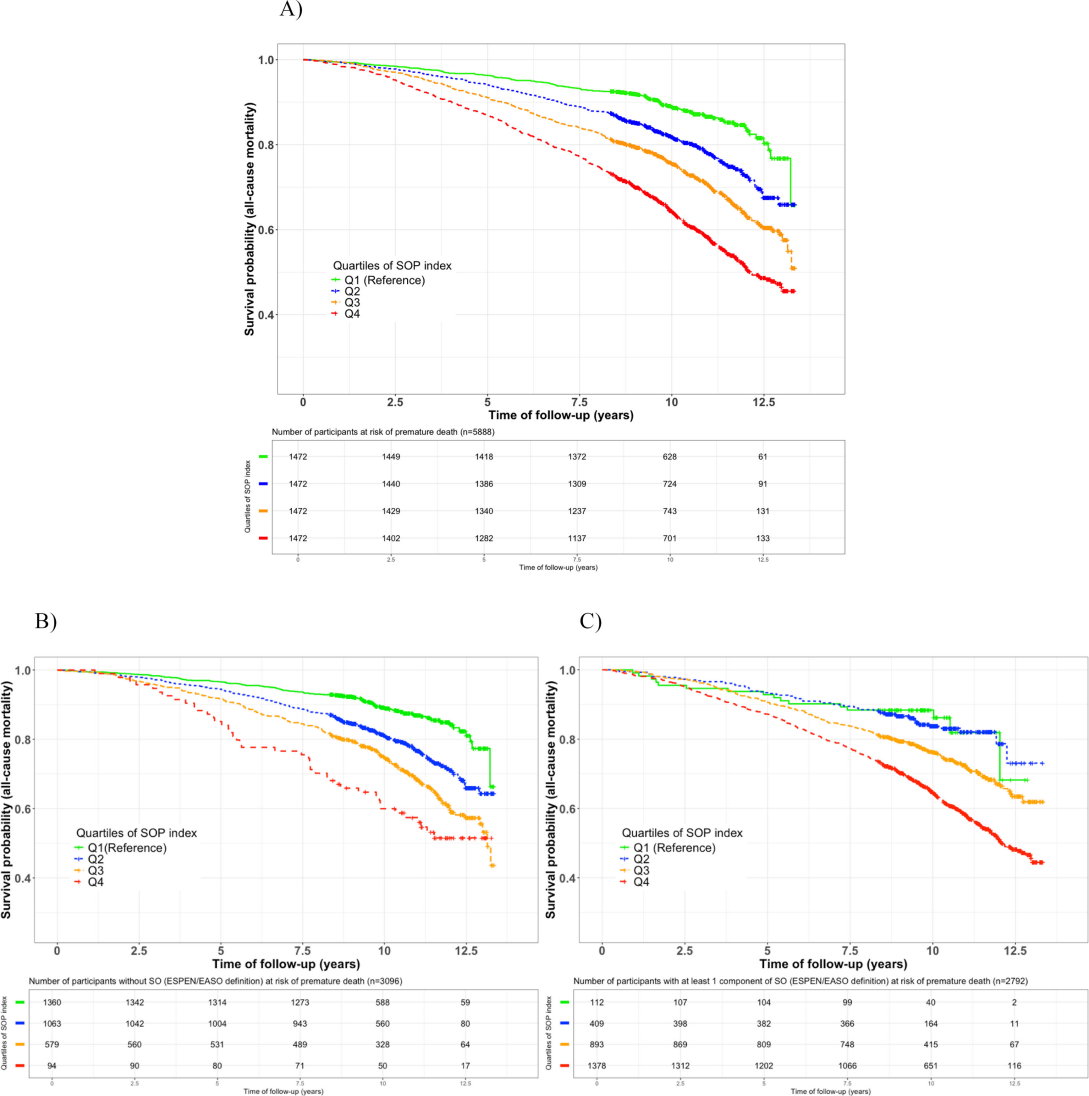
Kaplan–Meier curves for survival time for all‐cause mortality in (A) the whole population (*n* = 5888), (B) participants without any diagnostic criteria of SO (*n* = 3096) and (C) participants with at least one diagnostic criteria of SO (*n* = 2792).

During a median follow‐up of 9.9 years (IQR: 8.8–12.1), 1538 persons died. The proportional hazards assumption was found to be met. In the entire population, the highest quartile of SOP*i* was associated with an increased premature all‐cause mortality when compared to the lowest quartile (Figure 2; log‐rank *p* < 0.001).

**FIGURE 3:**
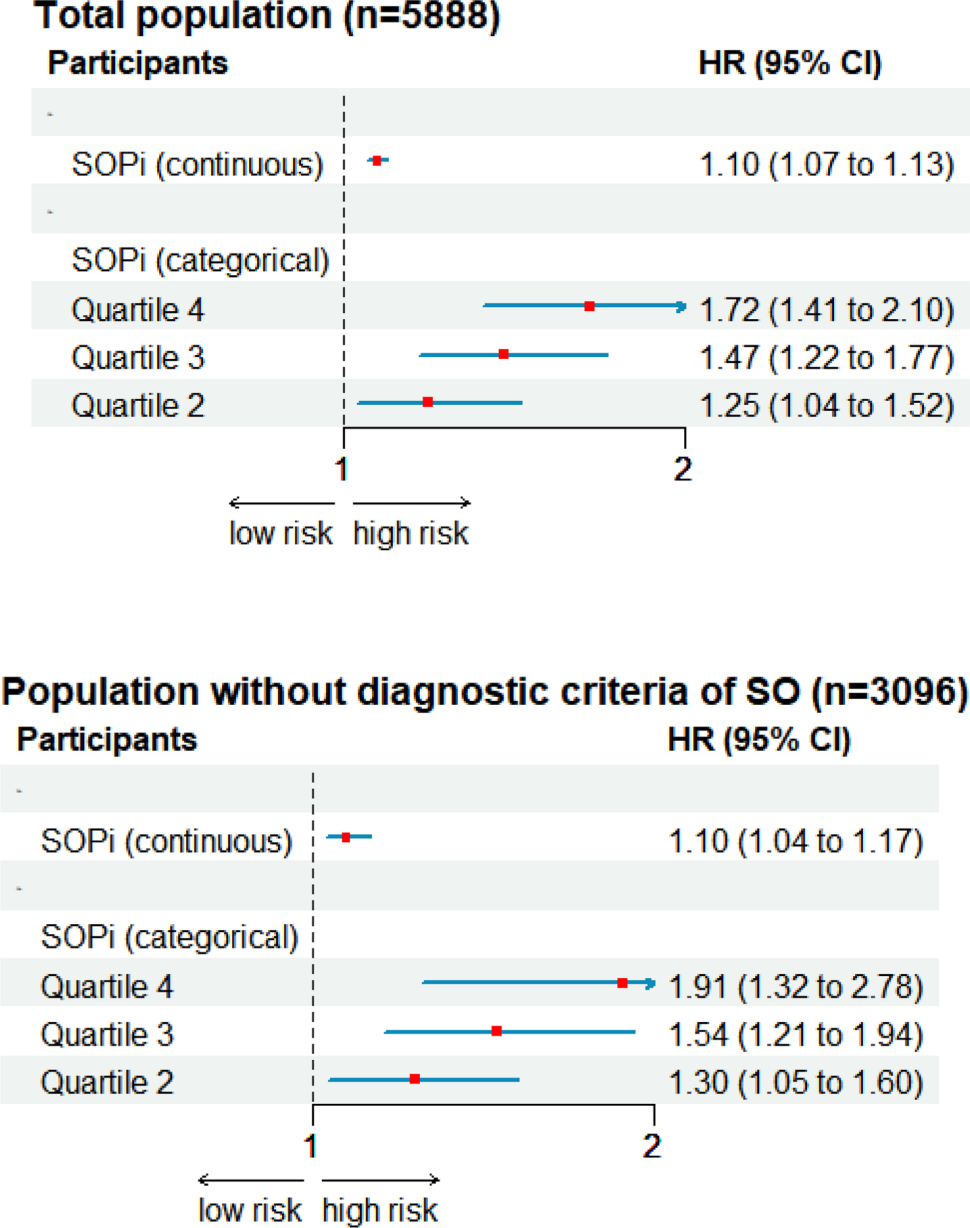
Association between SOP*i* and all‐cause mortality in (a) all population and (b) population without SO.

In Cox regression analyses, a higher SOP*i* was associated with worse survival probability in the entire population (HR = 1.10 [95%CI: 1.06; 1.13]). Likewise, in further analysis involving 3096 participants without any diagnostic criteria of SO, it was indicated that each SD of SOP*i* remained associated with mortality risk (HR = 1.11 [95%CI: 1.04; 1.17]). Furthermore, the highest quartile of SOP*i* continued to be highly predictive of premature death, even after adjustments for age, sex and BMI (Figure 3).

### 3.2 Factors Associated With SOP*i*

**FIGURE 4:**
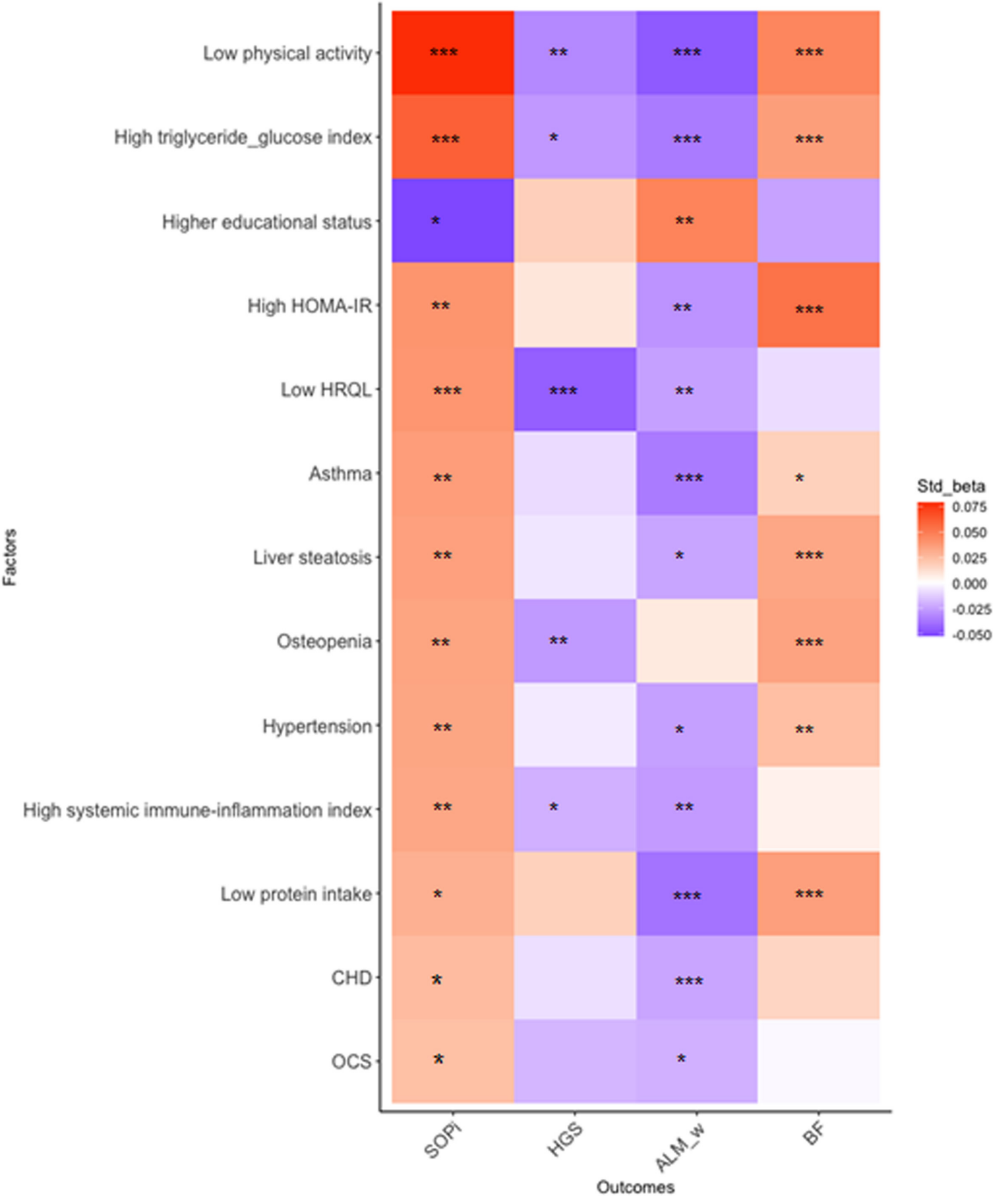
Heat map visualization of clinical and lifestyle factors associated with sex‐specific SOP*i* and its components in all population (*n* = 5888).

Of the 32 factors analysed individually using Model 1 (adjusted for age and BMI), 30 of them had a significant association with the sex‐specific SOP*i* (Table S2). The 30 factors were analysed in a multivariable model that included combined clinical factors (Model 2), combined lifestyle factors (Model 3) or all significant clinical and lifestyle factors from Models 2 and 3 combined (Model 4). In Model 5, thirteen factors remained significantly associated with sex‐specific SOP*i*, based on the significant factors identified in Model 4 (Figure 4 and Table S3).

A higher SOP*i* was associated with nine clinical factors: elevated metabolic (i.e., TyG and HOMA‐IR) and inflammatory markers (i.e., SII), comorbidities including liver steatosis, osteopenia, CHD, asthma and hypertension, as well as the use of OCS.

In addition, four lifestyle factors were associated with higher SOP*i*: reduced PA, low quality of life, low protein intake and low levels of education, as shown in Figure 4. Moreover, reduced PA and elevated TyG levels were the only two factors significantly associated with the three single components of SOP*i* (HGS, ALM/kg and BF%).

### 3.3 Longitudinal Changes of SOP*i*

A subset of 1682 participants who underwent a second SO examination was categorized into four groups according to the presence/absence of obesity and the two key significant associated factors indicated above: low PA and high TyG. Over a follow‐up period of 4.5 years [IQR: 4.34; 4.85], participants of the same age with obesity and one additional associated factor had an accelerated increase in their SOP*i* compared to participants without obesity and without associated factors (Figure S1). For instance, participants with obesity and 1 or more factors had a higher expected difference in SOP*i* (males: β = 2.63 [95%CI: 2.22; 3.03]; females: β = 2.90 [95%CI: 2.58; 3.23]) than those participants without obesity and 0 factors of the same age, and who were compared at the same follow‐up time (Table 2).

**TABLE 2:** Association between SOP*i* changes and study groups over the year.

	SOP*i* change per year			
Groups	Expected difference β [95%CI]			
	Males	*p*‐value	Females	*p*‐value
(1) Participants without obesity, 0 factors	Ref		Ref	
(2) Participants without obesity, 1 or more factors	0.71 [0.30; 1.13]	0.0007	0.69 [0.35; 1.03]	0.0001
(3) Participants with obesity, 0 factors	1.92 [1.37; 2.46]	< 0.001	2.24 [1.80; 2.67]	< 0.001
(4) Participants with obesity, 1 or more factors	**2.63 [2.22; 3.03]**	**< 0.001**	**2.90 [2.58; 3.23]**	**< 0.001**

### 3.4 Sensitivity Analyses

SOP*i* was linearly associated with all‐cause mortality, as indicated by the 5th and 95th percentile splines. In all populations, the logistic regression model yielded an AUC of 0.84 for the sex‐specific SOP*i*, adjusted for age, with a sensitivity of 74% and a specificity of 79% (Figure S2). In comparison to participants free of clinically diagnosed sarcopenic obesity, these values remain largely unchanged (Table S4). Table S5 represent percentiles, mean and SD of SOP*i* and its components for males and females, categorized by age group; the peak of SOP*i* was −1.92 ± 2.34 in males and −1.33 ± 2.44 in females in the younger age group (50–54 years). After excluding participants with BMI greater than 35 kg/m^2^ (*n* = 321), the associations of SOP*i* remained highly predictive of premature all‐cause mortality (Table S6).

## 4 Discussion

Results of this large population‐based study advocate a role for the continuous SOP*i* as a tool to identify persons at risk of SO and its subsequent health consequences, including premature death. This novel continuous SOP*i* was created by using the three ESPEN/EASO consensus SO diagnostic criteria: HGS, ALM adjusted by weight and body fat percentage. For each unit increase of SOP*i*, the total population and participants without any diagnostic criteria of SO had a lower 10‐year survival probability. When we divided the group into quartiles, participants with the highest SOP*i* (quartile 4) had worse survival probability compared to participants with the lowest SOP*i*. We identified several clinical and lifestyle factors associated with SOP*i*. Remarkably, over time, SOP*i* increases faster in persons with obesity, lower PA and/or insulin resistance.

Previous proposed scoring systems have identified persons with overweight or obesity at risk of SO [16, 33]. Particularly, Zambon and colleagues [16] developed a reliable diagnostic tool utilizing body composition measurements to identify cardiometabolic conditions in younger patients (mean age: 45 ± 12.9 years). Our study incorporated muscle function into the SOP*i*, which is a key SO diagnostic criterion, to improve early SO phenotypic detection. SOP*i* could be used as an additional tool to analyse risk stratification for all‐cause mortality, providing significant sensitivity and specificity compared to the existing dichotomous clinical SO definition. SOP*i* was applied to the entire population without anthropometric screening, and adiposity assessment was included in the index as a fat %. Some individuals who are classified as overweight (BMI > 25 and < 30 kg/m^2^) may not have excess body fat, while others with BMIs within the normal range may exhibit elevated percentages of fat [16, 31]. In older people, the current definition for obesity based on BMI may result in an underdiagnosis of obesity due to the loss of bone or muscle mass [16].

This novel approach to evaluate SO may be useful to assess the effectiveness of preventive or treatment interventions for SO. Similar to frailty continuous scores, SOP*i* may serve as a precise tool for identifying factors and biomarkers that hold clinical or functional significance for patients [18]. SOP*i* enables a more exact assessment of the dichotomous definition of SO, allowing researchers to examine the transition or progression of SO over time. Future research should externally validate this prognostic index alongside additional muscle function assessments such as chair stand and across different populations, including younger people or clinical‐based scenarios.

We examined the association of 32 preselected factors with SOP*i* and its components separately. SOP*i* was significantly associated with 13 factors. Both PA and TyG were associated with SOP*i* and its three components: HGS, ALM/w and BF%. Indeed, these two associated factors contributed to predicting a faster increase of SOP*i* in participants with obesity. Decreasing PA may accelerate age‐related muscle loss and function, reduce energy expenditure and raise the risk of obesity [1, 26]. A recent systematic review and meta‐analysis encompassing 11 RCTs on the effects of PA found that all the components of SO are improved [24]. The second factor, TyG, is a simple and reliable proxy of insulin resistance derived from triglycerides and plasma glucose. TyG does not require insulin measurement and may be used in all people. This finding suggests that insulin resistance plays a key role in the pathogenesis of SO [27]. Another classical factor associated with skeletal muscle health is protein intake. Recent recommendations of protein requirements in healthy adults, considering age‐related changes in metabolism, immunity, hormone synthesis, kidney function and progressive frailty, suggest a range of 0.8–1.2 g/kg per day [25]. In our study, daily protein intake was dichotomized as below, higher or equal to 1.0 g/kg, and it was associated with SOP*i*. Similarly, we found that daily protein intake was associated with ALM/kg and BF%, but not with HGS. Previous studies have shown similar results on lean and fat mass and inconclusive results on the continuous or dose–response effect of protein intake effect on HGS [20, 23, 24, 39].

The pathogenesis of SO, while not fully elucidated, involves elevated adipose deposits leading to the expression of antimyogenic adipokines and pro‐inflammatory responses that accelerate muscle function and/or mass loss. This promotes metabolic complications and inhibits the anabolic actions of insulin‐like growth factor IGF‐1, which have an effect on the musculoskeletal system [1, 21]. Elevated markers of insulin resistance (i.e., TyG and HOMA‐IR) and systemic inflammation (i.e., SII) were significantly associated with SOP*i* in our study. An in‐depth comprehension of the interplay between factors associated with the SOP*i*, its components and its phenotypes may help guide personalized weight loss treatments for patients with obesity. This understanding can help in the preservation of muscle mass and function through current and future pharmacological and nonpharmacological therapies [6, 40].

Our sensitivity analyses showed a linear association between SOP*i* and mortality, suggesting that the index displays predictable values across its range. However, the moderate discriminatory power observed in the ROC analysis highlights the need for additional refinement, potentially through the incorporation of functional, cardiometabolic or inflammatory biomarkers.

The strengths of this study include the use and the clinical assessment of the diagnostic criteria proposed by ESPEN/EASO Consensus and a long follow‐up. This study encompasses a large and comprehensive number of clinical and lifestyle factors, enabling us to examine their associations with the SOP*i*. Potential limitations should also be considered. First, clinical and lifestyle factors were assessed at baseline, and it is plausible that these factors might have changed over time. Second, due to the study population containing mainly people older than 60 years, a comparison with younger groups was not possible. Although older people face an increased risk of premature death due to ageing, we utilize age as a time scale to examine the differences of SOP*i* changes over 4.5 years of follow‐up. Finally, these results might be generalized to older Dutch people, and their applicability in other populations is anticipated.

## 5 Conclusion

In conclusion, a new continuous sex‐specific SOP*i* is proposed and validated for predicting the 10‐year mortality risk at a population level. Moreover, an accelerated SOP*i* increase over time was observed in participants with obesity, combined with lower levels of PA and/or insulin resistance. SOP*i* was associated with 13 clinical and lifestyle factors helping to define specific phenotypes. SOP*i* provides a clinically meaningful risk classification and, by targeting the associated factors, may serve as an effective and tailored approach for reducing the risk of SO. While the SOP*i* can capture the risk of premature death, it is important to assess its utility in clinical trials testing the response to pharmacological or nonpharmacological treatments.

## Conflicts of Interest

Elizabeth Benz, Alfonso J. Cruz‐Jentoft, Doris Eglseer, Eva Topinkova and Josje Schoufour reported receiving grants from the SO‐NUTS project, which is funded by JPI HDHL under the ERA‐NET cofund action No. 727565, outside the submitted work. Rocco Barazzoni disclosed his membership on advisory board panels for Novo Nordisk, Pfizer, Boehringer, Nutricia Research, as well as personal fees from Eli‐Lilly, unrelated to the submitted work. Yves Boirie disclosed receiving a grant from the Agence Nationale de la Recherche (ANR) during the conduct of the study and personal fees from Fresenius Kabi, Sanofi France, Novo Nordisk and Lilly outside the submitted work. Alexandre Pinel, Christelle Gillet, Frederick Capel, Bruno Pereira, Dimitris Rizopoulos, Lorenzo M. Donini, Fernando Rivadeneira, Marinka Steur, Trudy Voortman and Peter J.M. Weijs declared that they have no conflicts of interest.

## Supporting information


**Table S1:** Definition of SO according to ESPEN/EASO.
**Table S2:** Linear regression models for SOP*i* and the main associated factors.
**Table S3:** Standardized coefficients of factors associated with SOP*i* and its components (Model 5).
**Figure S1:** Longitudinal changes on the SOP*i*.
**Figure S2:** ROC curve of logistic regression model adjusted for age.
**Table S4:** Methods to calculate the cut‐off of SOP*i* and its components associated with all‐cause mortality.
**Table S5:** Percentiles of SOP*i* and its components.
**Table S6:** Association between SOP*i* and all‐cause mortality.
