# Supplementary material for: Sarcopenic Obesity Phenotype Index (SOPi): A Population‐Based Study
Source: J Cachexia Sarcopenia Muscle. 2025 Oct 15;16(5):e70099. doi: 10.1002/jcsm.70099 (PMC12528550; doi:10.1002/jcsm.70099)
Supplement: Supplementary file 1 — Table S1: Definition of SO according to ESPEN/EASO. Table S2: Linear regression models for SOPi and the main associated factors. Table S3: Standardized coefficients of factors associated with SOPi and its components (Model 5). Figure S1: Longitudinal changes on the SOPi. Figure S2: ROC curve of logistic regression model adjusted for age. Table S4: Methods to calculate the cut‐off of SOPi and its components associated with all‐cause mortality. Table S5: Percentiles of SOPi and its components. Table S6: Association between SOPi and all‐cause mortality. [file JCSM-16-e70099-s001.docx]

**Supplementary material**

Sarcopenic-obesity phenotype index (SOP*i*): a population-based study**.**

**Table of contents**

**Supplementary table 1**………………………………………………………………Page 3

Definition of SO according to ESPEN/EASO

**Supplementary table 2**………………………………………………………………Page 4

Linear regression models for SOP*i* and the main associated factors

**Supplementary table 3**………………………………………………………………Page 6

Standardized coefficients of factors associated with SOP *i* and its components (model 5)

**Supplementary figure 1**……………………………………………………………..Page 7

Longitudinal changes on the SOP*i*

**Supplementary figure 2**. ……………………………………………………………Page 8

ROC curve of logistic regression model adjusted for age

**Supplementary table 4**. …………………………………………………………….Page 9

Methods to calculate the cut-off of SOPi and its components associated with all-cause mortality

**Supplementary table 5**…………………………………………….………………..Page 10

Percentiles of SOPi and its components

**Supplementary table 6**…………………………………………….………………..Page 14

Association between SOP*i* and all-cause mortality

**Supplementary table 1.** Definition of SO according to ESPEN/EASO (1).

| Stages | Outcome | Cut-off values | |
| --- | --- | --- | --- |
| 1.Stage ONE  Muscle function | *Low muscle strength:*  measured by hydraulic hand dynamometer | **Male** | **Female** |
|  |  | <27 kg | <16 kg |
| 2. Stage TWO  Body composition | *Low muscle strength*  **AND**  *Low appendicular lean mass adjusted for weight*  measured by DXA.  **AND**  *High body fat percentage (BF%)*  measured by DXA. | <25.7%  Age: 40-59: >29%  Age: 60-79: >31% | <19.4%  Age: 40-59: >41%  Age: 60-79: > 43% |

**Abbreviations**: DXA: dual-energy X-ray absorptiometry. ESPEN/EASO: European Society for Clinical Nutrition and Metabolism/European Association for the Study of Obesity.

**Supplementary Table 2.** Linear regression models for sex-specific SOP*i* and the main associated factors.

| Factors | Model 1  β [95% CI] | Model 2  β [95% CI] | Model 3  β [95% CI] | Model 4  β [95% CI] | Model 5  β [95% CI] |
| --- | --- | --- | --- | --- | --- |
| **Anthropometric factors**  Age (years) | **-** | **0.09 [0.08; 0.10]** | **0.09 [0.08; 0.10]** | **0.09 [0.08; 0.10]** | **0.09 [0.08; 0.10]** |
| BMI (kg/m^2^) | **-** | **0.27 [0.26; 0.28]** | **0.29 [0.27; 0.30]** | **0.26 [0.24; 0.28]** | **0.26 [0.24; 0.28]** |
| **Clinical factors** |  |  |  |  |  |
| 1.TyG  1. <4.68  2. ≥4.68 | Ref  **0.39 [0.30; 0.49]** | Ref  **0.29 [0.18; 0.39]** | - | Ref  **0.27 [0.16; 0.38]** | Ref  **0.28 [0.16; 0.39]** |
| 2. HOMA-IR  1. <2.0  2. ≥2.0 | Ref  **0.39 [0.29; 0.49]** | Ref  **0.22 [0.11; 0.33]** | - | Ref  **0.19 [0.07; 0.32]** | Ref  **0.19 [0.07; 0.31]** |
| 3. SII index (sex-SII median)^a^ | **0.21 [0.13; 0.30]** | **0.15 [0.06; 0.24]** | - | **0.15 [0.04; 0.25]** | **0.15 [0.05; 0.25]** |
| 4. e GFR (<60 mL/min/1.73m^2^)  1. ≥60 mL/min/1.73m^2^  2. <60 mL/min/1.73m^2^ | Ref  0.09 [-0.04; 0.22] | - | - | - | - |
| 5. Dyslipidemia (sex-specific <HDL)^b^ | 0.11 [-0.00; 0.22] | - | - | **-** | **-** |
| 6. FEV1 % pred (<80)  1.≥ 80  2. <80 | **0.69 [0.56; 0.81]** | - | - | - | - |
| 7. Total BMD, g/cm^2^ | **-1.42 [-1.75; -1.10]** | - | - | - | - |
| Medication use (no vs yes)  8. OCS  9. Antidepressive therapy use | **0.67 [0.39; 0.94]**  **0.56 [0.36; 0.77]** | **0.62 [0.32; 0.92]**  **0.43 [0.21; 0.64]** | -  - | **0.36 [0.002; 0.71]**  0.25 [-0.01; 0.50] | **0.37 [0.01; 0.72]**  - |
| Comorbidities |  |  |  |  |  |
| 10. T2D  1. No  2. Yes | Ref  **0.18 [0.06;0.31 ]** | Ref  -0.02 [-0.15; 0.12] | - | - | - |
| 11. Osteopenia/osteoporosis  1. Normal  2. Osteopenia  3. Osteoporosis | Ref  **0.22 [0.12; 0.31]**  **0.41 [0.21; 0.60]** | Ref  **0.18 [0.08; 0.28]**  **0.34 [0.13; 0.56]** | - | Ref  **0.16 [0.05; 0.27]**  0.10 [-0.16; 0.36] | Ref  **0.16 [0.05; 0.27]**  - |
| 12. HT  1. No  2. Yes | Ref  **0.26 [0.15; 0.37]** | Ref  **0.18 [0.07; 0.30]** | - | Ref  **0.17 [0.05; 0.30]** | Ref  **0.17 [0.04; 0.30]** |
| 13. Chronic respiratory disease  1. No  2. COPD  3.Asthma | Ref  **0.21 [0.08; 0.34]**  **0.35 [0.18; 0.52]** | Ref  **0.14 [0.01; 0.27]**  **0.25 [0.08; 0.42]** | - | 0.14 [-0.01; 0.29]  **0.30 [0.11; 0.49]** | -  **0.31 [0.12; 0.50]** |
| 14. Cancer  1. No  2. Yes | Ref  **0.16 [0.01; 0.31]** | Ref  0.11 [-0.04; 0.26] | - | - | - |
| 15. Liver steatosis  1. No  2. Yes | Ref  **0.33 [0.23; 0.43]** | Ref  **0.20 [0.09; 0.31]** | - | Ref  **0.17 [0.05; 0.30]** | Ref  **0.17 [0.05; 0.30]** |
| 16. NALFD  1. No  2. Yes | Ref  **0.30 [0.19; 0.42]** | - | - | - | - |
| 17. MALFD  1. No  2. Yes | Ref  **0.31 [0.21; 0.41]** | - | - | - | - |
| 18. CHD  1. No  2. Yes | Ref  **0.29 [0.14; 0.45]** | Ref  **0.20 [0.03; 0.36]** | - | **0.21 [0.02; 0.40]** | **0.21 [0.02; 0.39]** |
| 19. Depression symptoms  1. CES-D <16  2. CES-D ≥16 | Ref  **0.19 [0.03; 0.34]** | Ref  0.02 [-0.14; 0.19] | - | - | - |
| **Lifestyle factors** |  |  |  |  |  |
| 1. Physical activity  1. ≥11.5 METh/w  2. <11.5 METh/w | Ref  **0.42 [0.33; 0.51]** |  | Ref  **0.38 [0.28; 0.48]** | Ref  **0.35 [0.25; 0.46]** | Ref  **0.36 [0.25; 0.46]** |
| 2. Total calorie intake, kcal/day | **-0.0003**  **[-0.0004; -0.0002]** | - | - | - | - |
| 3. Protein intake  1. ≥ 1.0 g/kg/day  2. <1.0 g/kg/day | Ref  **0.26 [0.14; 0.38]** |  | Ref  **0.17 [0.04; 0.30]** | Ref  **0.17 [0.04; 0.30]** | Ref  **0.16 [0.03; 0.30]** |
| 4. Fiber intake, g/day | **-0.02 [-0.03; -0.02**] | - | - | - | - |
| 5. Fat intake, g/day | **-0.003 [-0.004; -0.002]** | - | - | - | - |
| 6. Carbohydrate intake,g/day | **-0.002 [-0.003; -0.001]** | - | - | - | - |
| 7. Health-related quality of life^c^  1. ≥ 0.8  2. <0.8 | Ref  **0.43 [0.31; 0.54]** | - | **0.31 [0.16; 0.45]** | **0.24 [0.09; 0.39]** | **0.26 [0.11; 0.41]** |
| 8. Educational status  1. Primary education (ref)  2. Lower education  3. Intermediate education  4. Higher education | Ref  **-0.18 [-0.34; -0.01]**  **-0.25 [-0.42; -0.08]**  **-0.44 [-0.62; -0.27]** | - | Ref  -0.04 [-0.24; 0.17]  -0.15 [-0.36; 0.06]  **-0.29 [-0.51; -0.08]** | Ref  0.02 [-0.19; 0.23]  -0.10 [-0.32; 0.11]  **-0.27 [-0.49; -0.04]** | Ref  -  -  **-0.28 [-0.50; -0.05]** |
| 9. Sleep quality (tertiles of Index)^d^  1 Tertile 1  2 Tertile 2  3 Tertile 3 | -0.02 [-0.13; 0.10]  **0.13 [0.03; 0.24]** | - | -0.08 [-0.21;0.05]  0.05 [-0.06; 0.17] | - | - |
| 10. Alcohol intake, g | 0.003 [-0.02; 0.01] | - |  |  |  |
| 11. Smoking status  1. Never  2. Past  3. Current | Ref  0.090 [-0.01; 0.18]  -0.003 [-0.14; 0.15] | -  - |  |  |  |
| 12. Smoking pack years  1. <20  2. ≥20 | Ref  **0.16 [0.06; 0.26]** | - | Ref  0.05 [-0.06; 0.17] | - | - |
| 13. Retirement status  1. No  2. Yes | Ref  **1.84 [0.18; 3.50]** | - | - | - | - |

^a^ Sex-SII median: ≥458 in males and ≥463 in females

^b^ Dyslipidemia: HDL<1.0 mmol/L in males and<1.3 mmol/L in females

^c^ Low health-related quality of life: the median QoL EQ-5D-3L index was 0.8

^d^ Pittsburgh Sleep Quality Index: *tertile 1*: 1 ≤ 2, *tertile 2* between 2 and 5, and *tertile 3*: ≥ 5

Model 1: multivariable model adjusted for age and BMI and each factor.

Model 2: multivariable adjusted for age, BMI and all significant clinical factors from model 1 and VIF <2.

Model 3: multivariable model adjusted for age,BMI and all significant lifestyle factors from model 1 and VIF <2.0

Model 4: multivariable model adjusted for all the significant clinical and lifestyle factors from model 2-3 and VIF <2.0.

Model 5: multivariable model adjusted for all the significant factors from model 4

**Supplementary table 3:** Standardized coefficients of factors associated with SOP*i* and its components (model 5).

|  | Sex-specific SOPi |  | Components of SOPi | |  | |  | |
| --- | --- | --- | --- | --- | --- | --- | --- | --- |
|  |  |  | HGS (kg) | | ALM/w (kg/kg) | | BF% | |
| Factors | St b [95% CI] | p-value | St b [95% CI] | p-value | St b [95% CI] | p-value | St b [95% CI] | p-value |
| 1 BMI (kg/m^2^) | **0.4776 [0.4512; 0.5041]** | **<2.0×10^-16^** | **0.0401 [0.0168; 0.0634]** | **7.7×10^-04^** | **-0.3421 [-0.3641; -0.3201]** | **<2.0×10^-16^** | **0.5481 [0.5274; 0.5689]** | **<2.0×10^-16^** |
| 2 Age (years) | **0.3265 [0.3014; 0.3515]** | **<2.0×10^-16^** | **-0.3374 [-0.3595; -0.3161]** | **<2.0×10^-16^** | **-0.1780 [-0.1978; -0.1582]** | **<2.0×10^-16^** | **0.0257 [0.0074; 0.0440]** | **5.9×10^-03^** |
| 3 Physical activity | **0.0788 [0.0557; 0.1018]** | **2.7×10^-11^** | **-0.0324 [-0.0517; -0.0131]** | **1.0×10^-03^** | **-0.0465 [-0.0642; -0.0288]** | **2.8×10^-07^** | **0.0463 [0.0297; 0.0630]** | **5.0×10^-08^** |
| 4 TyG (1) | **0.0610 [0.0359; 0.0861]** | **2.1×10^-06^** | **-0.0276 [-0.0485; -0.0067]** | **1.0×10^-02^** | **-0.0362 [-0.0554; -0.0169]** | **2.4×10^-04^** | **0.0368 [0.0187; 0.0548]** | **6.8×10^-05^** |
| *5*.Higher education | **-0.0522 [-0.0944; -0.0100]** | **1.6×10^-02^** | 0.0180 [-0.0175, 0.0535] | 3.2×10^-01^ | **0.0470 [0.0144; 0.0796]** | **4.9×10^-03^** | -0.0246 [-0.0552; 0.0060] | 1.2×10^-01^ |
| *6*. HOMA-IR | **0.0402 [0.0142; 0.0663]** | **2.6×10^-03^** | 0.0092 [-0.0126; 0.0309] | 4.1×10^-01^ | **-0.0292 [-0.0492; -0.0092]** | **4.3×10^-03^** | **0.0539 [0.0351; 0.0727]** | **2.0×10^-08^** |
| *7*. Q of life | **0.0400 [0.0170; 0.0631]** | **6.8×10^-04^** | **-0.0448 [-0.0642; -0.0254]** | **6.5×10^-06^** | **-0.0252 [-0.0430, -0.0074]** | **5.7×10^-03^** | -0.0088 [-0.0255; 0.0079] | 3.0×10^-01^ |
| *8*. Asthma | **0.0372 [0.0141; 0.0603]** | **1.6×10^-03^** | -0.0094 [-0.0287; 0.0098] | 3.4×10^-01^ | **-0.0364 [-0.0541; -0.0187]** | **5.9×10^-05^** | **0.0172 [0.0006; 0.0338]** | **4.53×10^-02^** |
| 9. Liver steatosis | **0.0360 [0.0099; 0.0621]** | **7.0×10^-03^** | -0.0063 [-0.0281; 0.0154] | 5.7×10^-01^ | **-0.0241 [-0.0441; -0.0041]** | **1.9×10^-02^** | **0.0331[0.0144; 0.0519]** | **5.5×10^-04^** |
| 1*0*. Osteopenia | **0.0346 [0.0104; 0.0589]** | **5.3×10^-03^** | **-0.0271 [-0.0474; -0.0068]** | **9.1×10^-03^** | 0.0078 [-0.0109; 0.0264] | 4.2×10^-01^ | **0.0348 [0.0174; 0.0523]** | **9.9×10^-05^** |
| 1*1*. HT | **0.0337 [0.0085; 0.0589]** | **8.9×10^-03^** | -0.0055 [-0.0265; 0.0155] | 6.1×10^-01^ | **-0.0254[-0.0447; -0.0061]** | **1.0×10^-02^** | **0.0240 [0.0059; 0.0420]** | **9.7×10^-03^** |
| 1*2.SII (high)* | **0.0332 [0.0104; 0.0560]** | **4.5×10^-03^** | **-0.0210 [-0.0401; -0.0020]** | **3.1×10^-02^** | -**0.0269 [-0.0444; -0.0094]** | **2.7×10^-03^** | 0.0049 [-0.0115; 0.0212] | 5.6×10^-01^ |
| *13*. Daily protein intake | **0.0298 [0.0060; 0.0537]** | **1.5×10^-02^** | 0.0171 [-0.0028; 0.0370] | 9.4×10^-02^ | **-0.0388 [-0.0571; -0.0204]** | **5.06×10^-10^** | **0.0362 [0.0191; 0.0534]** | **3.8×10^-05^** |
| 14. CHD | **0.0255 [0.0023; 0.0487]** | **3.2×10^-02^** | -0.0085 [-0.0282; 0.0111] | 4.0×10^-01^ | **-0.0238 [-0.0419; -0.0058]** | **3.6×10^-05^** | 0.0155 [-0.0014; 0.0325] | 7.4×10^-02^ |
| 1*5*. OCS | **0.0235 [0.0007; 0.0463]** | **4.4×10^-02^** | -0.0191[-0.0381; 0.0000] | 5.0×10^-02^ | **-0.0209 [-0.0384; -0.0035]** | **1.9×10^-02^** | -0.0016 [-0.0180; 0.0148] | 8.5×10^-01^ |
| 1*6*. sex *(female)* | - | - | -0.7182 [-0.7361, -0.7002] | **<2.0×10^-16^** | **-0.6952 [-0.7135; -0.6768]** | **<2.0×10^-16^** | **0.5939 [0.5747; 0.6132]** | **<2.0×10^-16^** |
| R2 | 49% |  | 65% | | 70% | | 74% | |

**Supplementary figure 1.** Longitudinal changes on the SOP*i* according to the presence/absence of factors in participants with and without obesity.

**
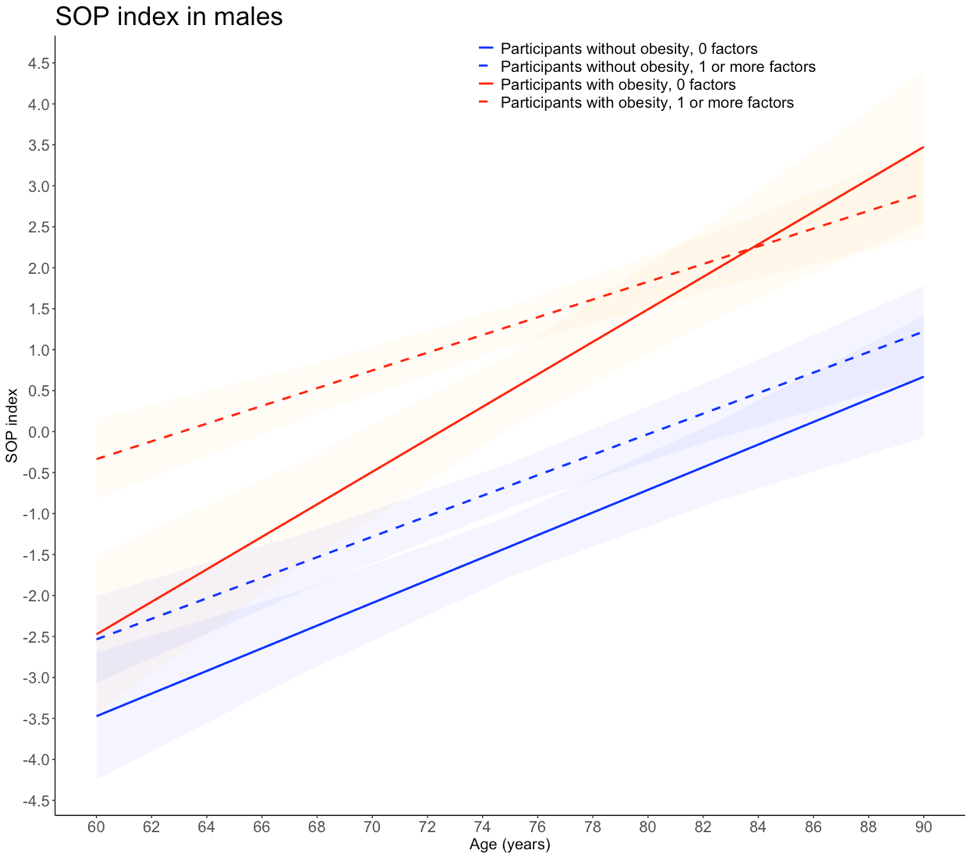
**


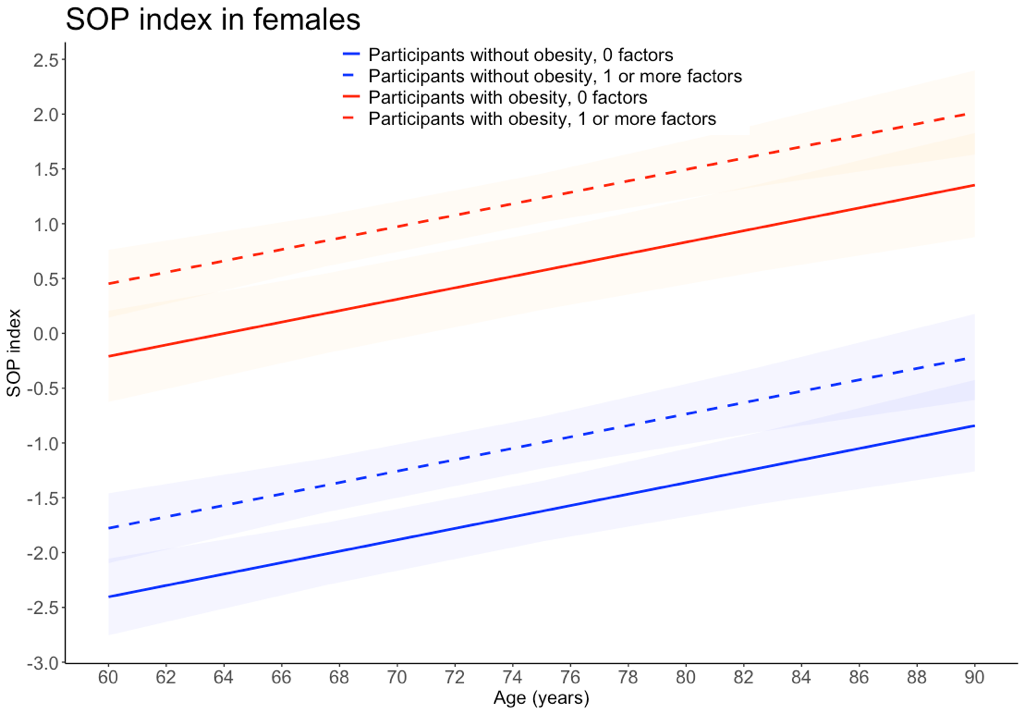


Plots represent the estimated differences in the SOP*i* when groups based on the presence/absence of obesity, lower physical activity and insulin resistance are reallocated into four groups (TyG): without obesity and without factors, without obesity with factors, with obesity without factors and with obesity with factors. Participants without obesity and without factors are used as reference. Solid colour lines represent the average SOP*i* trajectories over the years, with 95% CI (shadow colours).

**
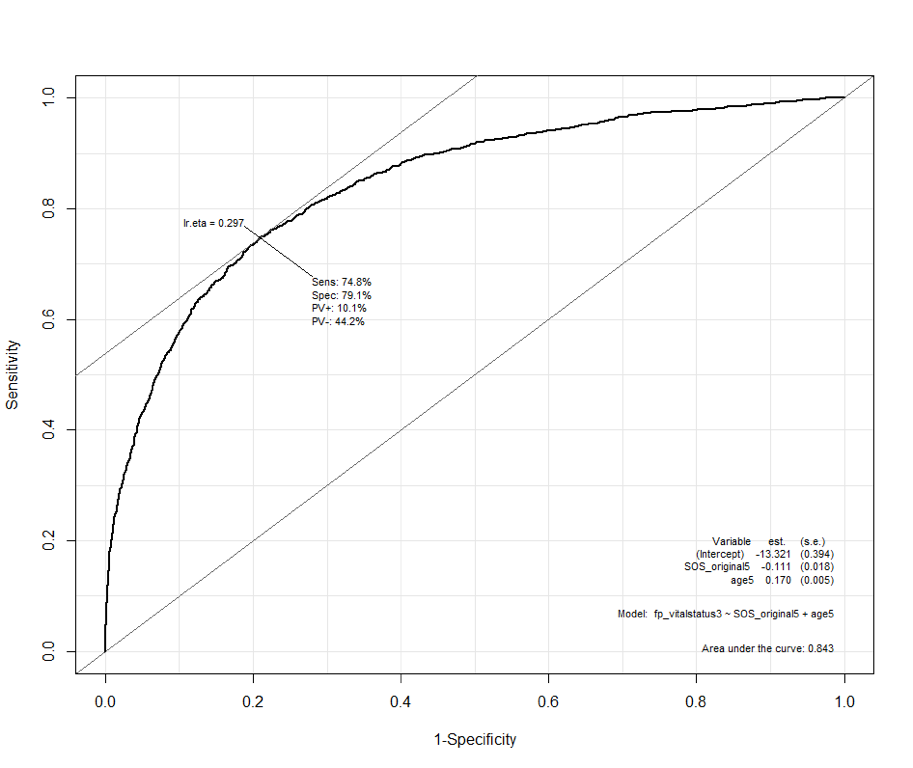

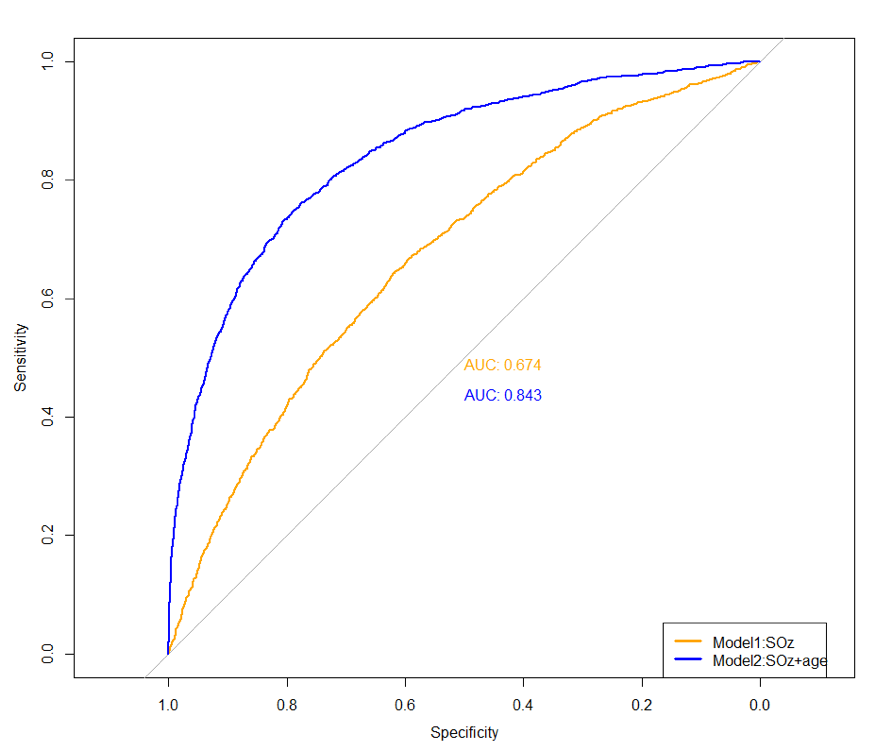
**

**Supplementary figure 2.** ROC curve of logistic regression model adjusted for age

**Supplementary table 4.** Methods to calculate the cut-off associated with all-cause mortality in all population.

| AUC | Optimal cut-off | Sensitivity (%) | Specificity (%) | AUC | Method |
| --- | --- | --- | --- | --- | --- |
| Male |  |  |  |  | Max.metric |
| BMI | 25 | 0.79 | 0.26 | 0.53 |  |
| Age | 72 | 0.76 | 0.76 | 0.83 |  |
| SOP*i* | 0.13 | 0.69 | 0.60 | 0.69 |  |
| HGS  ALM/w  Fat% | 35  31  29.4 | 0.68  0.49  0.68 | 0.68  0.79  0.40 | 0.74  0.68  0.54 |  |
| Female |  |  |  |  | Max. metric |
| BMI | 26 | 0.57 | 0.47 | 0.52 |  |
| age | 74 | 0.77 | 0.81 | 0.85 |  |
| SOP*i* | 0.39 | 0.65 | 0.61 | 0.66 |  |
| HGS  ALM/w  Fat% | 21  24.4  41 | 0.67  0.64  0.42 | 0.69  0.55  0.61 | 0.74  0.62  0.50 |  |

**Supplementary Table 5a**. Handgrip strength stratified by age groups in a) males and b) females (n=5888)

| Age (years) | n | **Handgrip strength** | | | | | | | |
| --- | --- | --- | --- | --- | --- | --- | --- | --- | --- |
| 1. **Males** |  | Percentiles | | | | | | | |
|  |  | **5th** | **10th** | **25th** | **50th** | **75th** | **90th** | **95^th^** | **Mean ±SD** |
| 50-54 | 130 | 30.45 | 34.00 | 40.00 | 44.50 | 50.00 | 55.00 | 57.10 | 44.61±8.43 |
| 55-59 | 314 | 29.00 | 32.00 | 36.00 | 42.00 | 48.00 | 52.00 | 56.00 | 42.30±8.20 |
| 60-64 | 373 | 26.00 | 30.00 | 35.00 | 40.00 | 45.00 | 49.00 | 52.80 | 39.91±8.20 |
| 65-69 | 586 | 25.00 | 28.00 | 33.00 | 38.00 | 42.00 | 47.00 | 50.00 | 37.81±7.57 |
| 70-74 | 437 | 24.00 | 27.00 | 30.00 | 35.00 | 40.00 | 44.00 | 48.00 | 35.31±7.26 |
| 75-79 | 382 | 21.00 | 23.00 | 27.00 | 32.00 | 36.00 | 41.00 | 44.00 | 31.86±7.15 |
| >80 | 323 | 16.00 | 19.20 | 24.00 | 27.00 | 32.50 | 38.00 | 40.00 | 28.12±6.97 |
| All | 2545 | 22.00 | 26.00 | 30.00 | 36.00 | 42.00 | 48.00 | 51.00 | 36.47±8.93 |

| Age (years) | n | **Handgrip strength** | | | | | | | |
| --- | --- | --- | --- | --- | --- | --- | --- | --- | --- |
| 1. **Females** |  | Percentiles | | | | | | | |
|  |  | **5th** | **10th** | **25th** | **50th** | **75th** | **90th** | **95^th^** | **Mean ±SD** |
| 50-54 | 165 | 18.00 | 20.00 | 24.00 | 26.00 | 30.00 | 32.00 | 34.00 | 26.42±4.91 |
| 55-59 | 413 | 17.00 | 19.00 | 22.00 | 26.00 | 29.00 | 31.00 | 34.00 | 25.47±5.16 |
| 60-64 | 495 | 17.00 | 19.00 | 22.00 | 24.00 | 27.00 | 30.00 | 32.00 | 24.39±4.70 |
| 65-69 | 722 | 15.00 | 17.00 | 20.00 | 23.00 | 26.00 | 29.00 | 32.00 | 23.09±4.97 |
| 70-74 | 585 | 13.00 | 15.00 | 18.00 | 21.00 | 24.00 | 27.00 | 30.00 | 21.03±5.14 |
| 75-79 | 478 | 11.00 | 13.00 | 16.00 | 19.00 | 22.00 | 25.00 | 27.00 | 19.13±4.70 |
| >80 | 485 | 9.00 | 10.00 | 13.00 | 16.00 | 19.00 | 22.00 | 24.00 | 16.20±4.57 |
| All | 3343 | 12.00 | 14.00 | 18.00 | 22.00 | 26.00 | 29.00 | 31.00 | 21.82±5.80 |

**Supplementary Table 5b**. Appendicular lean mass adjusted for weight (ALM/kg) stratified by age groups in a) males and b) females (n=5888).

| Age (years) | n | **ALM/kg** | | | | | | | |
| --- | --- | --- | --- | --- | --- | --- | --- | --- | --- |
| **a) Males** |  | Percentiles | | | | | | | |
|  |  | 5th | 10th | 25^th^ | 50th | 75th | 90th | 95^th^ | Mean ±SD |
| 50-54 | 130 | 27.18 | 28.38 | 30.84 | 32.65 | 34.75 | 36.71 | 37.72 | 32.59±3.25 |
| 55-59 | 314 | 27.67 | 28.48 | 30.06 | 31.73 | 33.93 | 35.35 | 36.58 | 32.99±2.94 |
| 60-64 | 373 | 26.85 | 27.66 | 29.36 | 31.27 | 33.16 | 34.80 | 35.80 | 31.26±2.80 |
| 65-69 | 586 | 26.01 | 26.87 | 28.51 | 30.52 | 32.36 | 34.17 | 35.37 | 30.50±2.90 |
| 70-74 | 437 | 25.53 | 26.56 | 28.08 | 29.88 | 31.64 | 33.06 | 34.01 | 29.84±2.66 |
| 75-79 | 382 | 25.06 | 25.95 | 27.70 | 29.26 | 30.98 | 32.72 | 33.74 | 29.33±2.56 |
| >80 | **323** | 24.80 | 25.39 | 26.95 | 28.85 | 30.59 | 31.94 | 33.42 | **28.81±2.67** |
| All | **2545** | 25.67 | 26.72 | 28.35 | 30.31 | 32.34 | 34.24 | 35.37 | 30.40±3.00 |

| Age (years) | n | **ALM/kg** | | | | | | | |
| --- | --- | --- | --- | --- | --- | --- | --- | --- | --- |
| **b)Females** |  | Percentiles | | | | | | | |
|  |  | 5th | 10th | 25^th^ | 50th | 75th | 90th | 95^th^ | Mean ±SD |
| 50-54 | 165 | 21.45 | 22.63 | 24.50 | 26.26 | 27.94 | 30.44 | 31.10 | 26.29±2.97 |
| 55-59 | 413 | 21.38 | 22.55 | 24.35 | 25.69 | 27.60 | 29.54 | 31.10 | 25.95±2.84 |
| 60-64 | 495 | 21.36 | 22.24 | 23.72 | 25.62 | 27.62 | 29.43 | 30.52 | 25.72±2.84 |
| 65-69 | 722 | 21.18 | 22.05 | 23.59 | 25.12 | 26.84 | 28.51 | 29.61 | 25.26±2.60 |
| 70-74 | 585 | 21.21 | 22.04 | 23.23 | 24.75 | 26.07 | 27.44 | 28.30 | 24.72±2.25 |
| 75-79 | 478 | 20.76 | 21.56 | 22.94 | 24.31 | 26.00 | 27.67 | 28.87 | 24.53±2.41 |
| **>80** | 485 | 20.75 | 21.57 | 22.73 | 24.16 | 25.77 | 27.68 | 28.87 | **24.42±2.43** |
| All | 3343 | 21.12 | 21.96 | 23.36 | 25.00 | 26.73 | 28.52 | 29.80 | 25.14±2.65 |

**Supplementary Table 5c**. Total body fat percentage stratified by age groups in a) males and b) females (n=5888).

| Age (years) | n | **BF%** | | | | | | | |
| --- | --- | --- | --- | --- | --- | --- | --- | --- | --- |
| 1. **Males** |  | Percentiles | | | | | | | |
|  |  | 5th | 10th | 25^th^ | 50th | 75th | 90th | 95^th^ | Mean ±SD |
| 50-54 | 130 | 21.23 | 23.48 | 25.84 | 28.95 | 32.94 | 36.29 | 40.58 | 29.53±5.72 |
| 55-59 | 314 | 21.53 | 23.01 | 27.03 | 30.13 | 34.06 | 37.21 | 38.59 | 30.18±5.61 |
| 60-64 | 373 | 22.21 | 24.17 | 27.37 | 30.85 | 34.64 | 37.45 | 39.41 | 30.87±5.37 |
| 65-69 | 586 | 21.68 | 24.33 | 27.77 | 31.37 | 35.08 | 38.33 | 40.41 | 31.36±5.59 |
| 70-74 | 437 | 22.29 | 24.50 | 27.69 | 31.43 | 35.04 | 38.81 | 40.23 | 31.39±5.52 |
| 75-79 | 382 | 22.17 | 24.66 | 28.00 | 31.48 | 34.80 | 38.17 | 40.21 | 31.36±5.43 |
| >80 | 323 | 22.05 | 24.66 | 28.28 | 31.57 | 35.48 | 37.79 | 39.77 | 31.37±5.43 |
| All | 2545 | 21.93 | 24.17 | 27.49 | 31.11 | 34.81 | 37.98 | 40.01 | 31.06±5.53 |

| Age (years) | n | **BF%** | | | | | | | |
| --- | --- | --- | --- | --- | --- | --- | --- | --- | --- |
| 1. **Females** |  | Percentiles | | | | | | | |
|  |  | 5th | 10th | 25th | 50th | 75th | 90th | 95^th^ | Mean ±SD |
| 50-54 | 165 | 26.65 | 30.11 | 34.35 | 38.73 | 43.47 | 48.05 | 50.34 | 38.72±6.99 |
| 55-59 | 413 | 27.73 | 30.64 | 34.78 | 38.80 | 43.30 | 46.72 | 48.98 | 38.79±6.49 |
| 60-64 | 495 | 28.87 | 30.61 | 35.28 | 39.32 | 43.73 | 47.30 | 48.73 | 39.23±6.29 |
| 65-69 | 722 | 30.18 | 32.23 | 35.71 | 39.27 | 43.15 | 46.82 | 49.54 | 39.41±5.82 |
| 70-74 | 585 | 30.43 | 32.57 | 36.63 | 40.03 | 43.34 | 46.34 | 48.12 | 39.79±5.43 |
| 75-79 | 478 | 29.14 | 31.55 | 36.05 | 40.24 | 44.05 | 47.49 | 48.83 | 39.78±6.16 |
| >80 | 485 | 28.26 | 31.37 | 35.04 | 39.15 | 42.94 | 46.55 | 48.24 | 38.94±6.17 |
| All | 3343 | 29.09 | 31.59 | 35.50 | 39.51 | 43.46 | 46.97 | 48.97 | 39.32±6.08 |

**Supplementary Table 5d.** Sex-specific sarcopenic obesity phenotype index stratified by age groups in a) males and b) females (n=5888).

| Age (years) | n | SOPi | | | | | | | |
| --- | --- | --- | --- | --- | --- | --- | --- | --- | --- |
| **a) Males** |  | Percentiles | | | | | | | |
|  |  | 5th | 10th | 25^th^ | 50th | 75th | 90th | 95^th^ | Mean ±SD |
| 50-54 | 130 | -5.79 | -4.85 | -3.50 | -1.85 | -0.50 | 1.33 | 1.62 | -1.92±2.34 |
| 55-59 | 314 | -5.03 | -3.89 | -2.72 | -1.27 | 1.17 | 1.31 | 1.75 | -1.34±2.09 |
| 60-64 | 373 | -4.24 | -3.48 | -2.01 | -0.76 | 0.81 | 1.76 | 2.68 | -0.71±2.12 |
| 65-69 | 586 | -3.60 | -3.14 | -1.59 | -0.17 | 1.40 | 2.79 | 3.47 | -0.13±2.21 |
| 70-74 | 437 | -3.02 | -2.45 | -1.16 | 0.48 | 1.72 | 3.10 | 3.82 | 0.38±2.14 |
| 75-79 | 382 | -2.27 | -1.69 | -0.43 | 0.94 | 2.30 | 3.19 | 4.13 | 0.93±1.98 |
| >80 | 323 | -1.66 | -1.06 | 0.28 | 1.63 | 2.97 | 3.96 | 4.53 | 1.52±1.99 |
| All | 2545 | -3.85 | -3.06 | -1.53 | 0.06 | 1.57 | 2.95 | 3.68 | 0.00 |

| Age (years) | n | SOPi | | | | | | | |
| --- | --- | --- | --- | --- | --- | --- | --- | --- | --- |
| **b)Females** |  | Percentiles | | | | | | | |
|  |  | 5th | 10th | 25^th^ | 50th | 75th | 90th | 95^th^ | Mean ±SD |
| 50-54 | 165 | -5.47 | -4.45 | -2.92 | -0.99 | 0.12 | 1.64 | 2.61 | -1.33±2.44 |
| 55-59 | 413 | -5.01 | -4.06 | -2.47 | -0.95 | 0.33 | 1.90 | 2.79 | -1.02±2.35 |
| 60-64 | 495 | -4.53 | -3.73 | -2.29 | -0.66 | 0.97 | 2.30 | 2.89 | -0.68±2.32 |
| 65-69 | 722 | -3.64 | -2.90 | -1.71 | -0.21 | 1.12 | 2.51 | 3.32 | -0.25±2.18 |
| 70-74 | 585 | -2.85 | -1.99 | -0.93 | 0.44 | 1.63 | 2.71 | 3.52 | 0.37±1.96 |
| 75-79 | 478 | -2.97 | -1.76 | -0.47 | 0.97 | 2.11 | 3.21 | 3.78 | 0.77±2.01 |
| >80 | 485 | -2.38 | -1.40 | -0.12 | 1.39 | 2.52 | 3.52 | 4.01 | 1.18±1.95 |
| All | 3343 | -3.97 | -2.94 | -1.44 | 0.11 | 1.59 | 2.78 | 3.56 | 0.00 |

**Supplementary table 6.** Association between SOP index and all-cause mortality

|  | SOP*i* (continuous)  HR [95% CI] | SOP*i* (categorical)  HR [95% CI] | | |
| --- | --- | --- | --- | --- |
| **All participants (n=5888)** | SOP index | Q1 vs Q4 | Q1 vs Q3 | Q1 vs Q2 |
| Model 0 | **1.23 [1.20; 1.26]** | **3.72 [3.14; 4.41]** | **2.47 [2.07; 2.95]** | **1.75 [1.46; 2.12]** |
| Model 1 | **1.07 [1.04; 1.10]** | **1.57 [1.32; 1.88]** | **1.40 [1.17; 1.67]** | **1.24 [1.03; 1.50]** |
| Model 2 | **1.10 [1.07; 1.13]** | **1.76 [1.44; 2.14]** | **1.49 [1.23; 1.80]** | **1.29 [1.06; 1.56]** |
| Model 3 | **1.10 [1.06; 1.13]** | **1.75 [1.43; 2.13]** | **1.46 [1.20; 1.76]** | **1.30 [1.08; 1.58]** |
| *a) Participants without any diagnostic criteria of SO (n=3096)* |  |  |  |  |
| Model 0 | **1.28 [1.22; 1.35]** | **3.75 [2.67; 5.26]** | **2.69 [2.18; 3.33]** | **1.89 [1.54; 2.31]** |
| Model 1 | **1.08 [1.03; 1.14]** | **1.82 [1.28; 2.58]** | **1.44 [1.15; 1.80]** | **1.26 [1.03; 1.55]** |
| Model 2 | **1.10 [1.04; 1.17]** | **1.99 [1.38; 2.89]** | **1.55 [1.22; 1.96]** | **1.32 [1.07; 1.63]** |
| Model 3 | **1.11 [1.04; 1.17]** | **2.00 [1.39; 2.90]** | **1.56 [1.23; 1.98]** | **1.34 [1.08; 1.66]** |
| *b) Participants with at leat 1 diagnostic criteria of SO (n=2792)* |  |  |  |  |
| Model 0 | **1.23 [1.19; 1.28]** | **2.79 [1.69; 4.58]** | **1.74 [1.05; 2.88]** | 1.08 [0.63; 1.87] |
| Model 1 | **1.07 [1.02; 1.11]** | 1.09 [0.66; 1.81] | 0.95 [0.57; 1.58] | 0.86 [0.50; 1.49] |
| Model 2 | **1.10 [1.05; 1.16]** | 1.21 [0.72; 2.01] | 1.01 [0.61; 1.68] | 0.89 [0.51; 1.54] |
| Model 3 | **1.10 [1.05; 1.15]** | 1.22 [0.73; 2.04] | 0.99 [0.59; 1.65] | 0.91 [0.52; 1.58] |
|  |  |  |  |  |
| **Participants with BMI <35 *kg/m^2^* (n=5567)** |  |  |  |  |
| Model 0 | **1.26 [1.23; 1.29]** | **4.00 [3.37; 4.75]** | **2.53 [2.11; 3.01]** | **1.75 [1.45; 2.11]** |
| Model 1 | **1.07 [1.04; 1.10]** | **1.53 [1.28; 1.83]** | **1.38 [1.15; 1.65]** | **1.21 [1.00; 1.46]** |
| Model 2 | **1.11 [1.07; 1.14]** | **1.80 [1.47; 2.20]** | **1.54 [1.27; 1.86]** | **1.29 [1.06; 1.56]** |
| Model 3 | **1.10 [1.07; 1.14]** | **1.80 [1.47; 2.20]** | **1.50 [1.24; 1.82]** | **1.30 [1.07; 1.58]** |

**Model 0**: unadjusted; **Model 1**: model adjusted for age and sex ; **Model 2**: model 1+ BMI; **Model 3**: model 2+ smoking status + number comorbidities.
